# New model for diabetes primary health care based on patient empowerment and the right to preventive health: the MIDE program

**DOI:** 10.26633/RPSP.2017.128

**Published:** 2017-11-30

**Authors:** Margarita Blanco-Cornejo, Irma Luz Riva-Palacio-Chiang-Sam, Iyari Sánchez-Díaz, Antonio Cerritos, Carlos Tena-Tamayo, Daniel López-Hernández

**Affiliations:** 1 Health Protection and Prevention Sub-Directorate Medical Directorate, Instituto de Seguridad y Servicios Sociales de los Trabajadores del Estado Mexico City Mexico Health Protection and Prevention Sub-Directorate, Medical Directorate, Instituto de Seguridad y Servicios Sociales de los Trabajadores del Estado, Mexico City, Mexico.; 2 Hospital Regional de Alta Especialidad del Bajío Ministry of Health León, GJ Mexico Hospital Regional de Alta Especialidad del Bajío, Ministry of Health, León, GJ, Mexico.

**Keywords:** Primary health care, health services, health promotion, delivery of health care, health care (public health), health policy, Mexico, Americas, Atención primaria de salud, servicios de salud, promoción de la salud, asistencia sanitaria, atención de salud, política de salud, México, Américas, Atenção primária à saúde, serviços de saúde, promoção da saúde, assistência à saúde, atenção à saúde, política de saúde, México, Américas

## Abstract

**Objective.:**

*To evaluate Mexico’s national Integrated Management of Diabetes in Stages (*Manejo Integral de la Diabetes por Etapas, *MIDE) program using three types of indicators: process, structure, and impact*.

**Methods.:**

*A cross-sectional study was conducted using data for 97 452 people with diabetes (PWD) who participated in the MIDE patient empowerment program (PEP) at “MIDE modules” (standardized diabetes health care units) at Mexico’s Institute for Social Security and Services for State Workers* (Instituto de Seguridad y Servicios Sociales de los Trabajadores del Estado, *ISSSTE) hospital clinics and family medical clinics nationwide between 2007 and 2014. The program promotes diabetes patient empowerment and self-care through outpatient consultations with a multidisciplinary health care team supported by continuous training. Baseline data were compared with results post-program for the following indicators: process (metabolic control (MetC), based on glycated hemoglobin (HbA1c), triglyceride (TG), and total cholesterol (TC) levels); structure (number of MIDE modules installed at ISSSTE clinics and number of patients/health personnel accredited as diabetes experts/awarded diplomas); and impact (average number of patient illness days (IDs) and hospitalization episodes (HEs) per PWD over a 12-month period)*.

**Results.:**

*Over the seven-year study period, the proportion of patients with MetC (HbA1c < 7.0%, TG < 150 mg/dL, and TC < 200 mg/dL) increased significantly (from 35.4% to 60% (with a peak level of 62% in 2013); P < 0.001); average HbA1c, triglycerides, and total cholesterol per PWD dropped by 25%, 31%, and 11% respectively; average number of IDs and HEs per PWD over a 12-month period dropped by 38% and 41% respectively; a total of 140 MIDE modules were installed at ISSSTE clinics; and a total of 1 117 diplomas were awarded to 826 health professionals, and 2 613 PWD were accredited as “patient experts in diabetes*.”

**Conclusions.:**

*The MIDE PEP is feasible, usable, and acceptable to PWD. The program improves MetC; reduces the frequency of IDs and HEs; and facilitates patient participation, the involvement of health personnel, and shared decision-making*.

## RESUMEN

**Objetivo.:**

Evaluar el programa nacional mexicano “Manejo Integral de la Diabetes por Etapas” (MIDE) usando tres tipos de indicadores: proceso, estructura e impacto.

**Métodos.:**

Se realizó un estudio transversal con los datos de 97 452 personas con diabetes que participaron entre el 2007 y el 2014 en el programa de empoderamiento de pacientes de MIDE que se llevó a cabo en los “módulos del MIDE” (unidades estandarizadas de atención de salud de diabetes) en el Instituto de Servicios y de Seguridad Sociales de los Trabajadores del Estado (ISSSTE) de México, además de en consultorios de hospitales y centros de medicina familiar de todo el país. El programa promueve el empoderamiento y el autocuidado en los pacientes con diabetes mediante consultas ambulatorias con un equipo de atención de salud multidisciplinario complementadas con talleres de capacitación continua. Se compararon los datos iniciales y los resultados al final del programa con respecto a los siguientes indicadores: proceso (control metabólico basado en análisis de los niveles de glucohemoglobina [HbA1c], triglicéridos y colesterol total); estructura (número de módulos del MIDE instalados en clínicas del ISSSTE y número de pacientes y personal de salud acreditado como experto en diabetes o que ha obtenido un certificado); e impacto (número promedio de días enfermos de los pacientes y número de hospitalizaciones por persona con diabetes durante un período de 12 meses).

**Resultados.:**

En el período de estudio de siete años, la proporción de pacientes con control metabólico (HbA1c < 7,0%, triglicéridos < 150 mg/dL y colesterol total < 200 mg/dL) aumentó significativamente (de 35,4% a 60%, con un nivel máximo de 62% en 2013; P < 0,001); el nivel promedio de HbA1c, triglicéridos y colesterol total por persona con diabetes descendió en 25%, 31% y 11% respectivamente; el número promedio de días enfermos y número de hospitalizaciones por persona con diabetes durante un período de 12 meses descendió en 38% y en 41% respectivamente; se instalaron un total de 140 módulos del MIDE en clínicas del ISSSTE, y se otorgaron un total de 1117 certificados a 826 profesionales de la salud y 2613 personas con diabetes fueron acreditados como “paciente experto en diabetes”.

**Conclusiones.:**

El programa de empoderamiento de pacientes del MIDE es un programa factible y aceptable para utilizarse con las personas con diabetes. El programa mejora los valores del control metabólico; reduce la frecuencia de días enfermos y número de hospitalizaciones; y facilita la participación tanto de los pacientes como del personal de salud, así como la toma de decisiones compartida.

## RESUMO

**Objetivo.:**

Avaliar o Programa de manejo integral de diabetes por etapas (MIDE) no México segundo três tipos de indicadores: processo, estrutura e impacto.

**Métodos.:**

Estudo transversal realizado com dados de 97.452 pessoas com diabetes (PD) que participaram do Programa MIDE de empoderamento do paciente em “módulos” designados (unidades de saúde que prestam atenção padronizada à diabetes) de ambulatórios hospitalares e centros de medicina da família do Instituto de Serviços e Seguridade Social dos Trabalhadores do Estado (ISSSTE) entre 2007 e 2014. O programa promove o empoderamento e o autocuidado dos pacientes com diabetes realizando consultas ambulatoriais com uma equipe de saúde multidisciplinar que recebe capacitação contínua. Os dados de referência foram comparados aos resultados pós-programa para os seguintes indicadores: processo (controle metabólico segundo os níveis de hemoglobina glicada [HbA1c], triglicerídeos [TG] e colesterol total [CT]); estrutura (número de módulos do MIDE instalados nos ambulatórios do ISSSTE e número de pacientes/pessoal de saúde credenciados como especialistas/diplomados em diabetes) e impacto (número médio de dias de doença do paciente [DD] e internações hospitalares (IH) por PD por um período de 12 meses).

**Resultados.:**

No período de estudo de sete anos, o percentual de pacientes com controle metabólico (HbA1c <7,0%, TG <150 mg/dl e CT <200 mg/dl) aumentou significativamente (de 35,4% a 60%, atingindo o ponto mais alto de 62% em 2013; P < 0,001). Houve redução dos níveis médios de HbA1c, TG e CT por PD de 25%, 31% e 11%, respectivamente. O número médio de DD e IH por PD em um período de 12 meses teve queda de 38% e 41%, respectivamente. Ao todo, foram instalados 140 módulos do Programa MIDE nos ambulatórios do ISSSTE, 1.117 diplomas foram concedidos a 826 profissionais da saúde e 2.613 PD foram credenciados como “pacientes especialistas em diabetes”.

**Conclusões.:**

O programa MIDE de empoderamento do paciente é viável, utilizável e aceitável às PD. Promove melhor controle metabólico da diabetes, reduz o número de DD e IH e facilita a participação de pacientes, o envolvimento do pessoal da saúde e a tomada de decisão conjunta.

At all levels of health care, most patients are treated under the “medicalization of medical care” paradigm^3^ (1). The classical focus of this model of medical care transfers full responsibility for the medical problem or condition, including those affected by patient behavior and lifestyle, to the public health system. Several studies report that a person’s ability to develop and acquire cognitive behavioral tools that promote health (“patient empowerment”) is essential to 1) procure and encourage self-care and medical adherence and 2) avoid complications resulting from noncommunicable diseases (NCDs) (2–10). In people with diabetes (PWD), patient empowerment has been shown to improve 1) various indicators for metabolic control (MetC), such as glycated hemoglobin (HbA1c), blood pressure, and low-density lipoprotein cholesterol (2, 6), and 2) other process indicators for disease management, such as knowledge and self-efficacy, communication with the physician, and quality of life. As patient empowerment has also been shown to help control microvascular and renal diseases, several studies of those medical conditions have incorporated person-centered medical care (4–10). These outcomes suggest the need to develop new strategies and health care policies that incorporate multidimensional solutions to complex public health issues (11).

## FOCUS ON PRIMARY HEALTH CARE AT ISSSTE

To address the need for a new, more integrated approach to public health, Mexico’s Institute for Social Security and Services for State Workers (*Instituto de Seguridad y Servicios Sociales de los Trabajadores del Estado*, ISSSTE) developed a new model for primary health care, focused on diabetes—Integrated Management of Diabetes in Stages *(Manejo Integral de la Diabetes por Etapas*, MIDE). In this model, education of both the patients and health professionals is the backbone of prevention strategies that are designed to help 1) generate a culture of patient self-care (2, 11) and responsibility, and 2) increase the quality of preventive health care services. This new paradigm for health care, based on patient empowerment, preventive care, training of health personnel, and strengthening of the health care system (11), could gradually reverse the incidence of diabetes and other NCDs.

### MIDE model

The MIDE model is based on five basic elements: 1) preventive care; 2) empowerment of the patient and his/her family; 3) continuous training for health care personnel; 4) adequate facilities, medicines, medical supplies, and medical equipment in primary health care units; and 5) efficient use of information and communication technologies. The model also incorporates strategies to integrate different program components and encourage the participation of the patient’s family and social networks.

### MIDE program

In 2007, the MIDE model was put into practice as an ISSSTE preventive medicine program that focuses on developing cognitive-behavioral skills and capabilities to promote active participation by patients, their family members, and their social networks; health care personnel; and health care institutions. This preventive care PEP (patient empowerment program) is carried out through outpatient consultations with a multidisciplinary health care team at standardized diabetes health care units (“MIDE modules”) installed at ISSSTE hospital clinics and family medical clinics (*Clínicas hospital y clínicas de medicina familiar*) nationwide. Continuous training for both patients and health personnel is provided through structured education programs.

#### Patient entry and exit criteria.

Any PWD can be assessed and treated at the MIDE modules. Entry criteria are as follows: preprandial blood glucose > 130 mg/dL or < 70 mg/dL; postprandial glucose > 180 mg/dL; and HbA1c > 7%. Meeting one criterion is sufficient for admission into the program. If the patient leaves the treatment program or is absent from the outpatient medical consultation for six months or longer, he/she is considered to have left the program. If the patient dies from causes related or unrelated to diabetes, he/she is recorded as leaving the program due to death. Each patient attends the medical module for one year. When the patient meets the criteria for a favorable response to the integrated treatment, he/she continues to be followed-up at the same medical unit with her/his physician, and visits the module every six months for review of his/her MetC. A patient is considered to have a positive response to the integrated treatment if he/she has reached an acceptable level of empowerment (according to the program’s accreditation criteria), and presents an optimal MetC, as shown in at least two determinations of HbA1c as < 7% (taken three months apart). Patients with sustained uncontrolled MetC and/or detected complications are referred to a secondary level of health care.

#### Accreditation criteria.

Program accreditation criteria are summarized in Supplementary Material Tables 1, 2, and 3. A score of 80 or higher (on a scale of 0–100 points) is required to obtain MIDE accreditation.

The objective of this study was to evaluate the success of the MIDE program using three types of indicators: process, structure, and impact.

## MATERIAL AND METHODS

### Protocol and study population

A cross-sectional study was conducted using MIDE patient data for January 2007 to December 2014 from the national MIDE database. All 97 452 MIDE participants included in the study provided a structured clinical history.

Baseline data was compared with post-program results for the following indicators: process (MetC), based on HbA1c, triglyceride, and total cholesterol levels); structure (number of MIDE modules installed at ISSSTE clinics and number of patients/health personnel accredited as diabetes experts/awarded diplomas); and impact (average number of patient illness days (IDs) and hospitalization episodes (HEs) per PWD over a 12-month period).

Based on changes in these indicators over the 12-month program participation period, four main program components were evaluated: 1) the MIDE modules (quality, and quantity installed); 2) patient care (multidisciplinarity and integration of services provided, including consultations with nurses, psychologists, nutritionists, and dental and physical activity specialists); 3) education program (number of patients/health personnel that were trained/accredited); and 4) medical outcomes (MetC and decrease in IDs and HEs).

### MIDE modules

To accomplish the main objective of providing medical care, a medical module (the “MIDE module”) was designed to operate mainly in primary health care units (only a few modules are located in secondary and tertiary health care units). Each medical module has 1) a computer with an Internet connection, to maintain records for medical histories and subsequent outpatient medical consultations, and 2) reagents and equipment, for quantification of capillary HbA1c and detection of microalbuminuria. The physician assigned to the module provides an initial outpatient consultation and a minimum of three subsequent outpatient consultations for each patient. These four+ consultations are designed to ensure high-quality medical care while promoting patient empowerment. During the initial outpatient consultation, the physician determines the capillary levels of HbA1c microalbuminuria and requests complementary laboratory studies (a lipid profile). Through this process, a baseline health status is established for use in monitoring/evaluating the health outcomes of each patient, and a treatment is specified, based on the recommendations of the International Diabetes Federation (IDF) Clinical Guidelines Task Force (12). The four main outpatient consultations also provide an opportunity to 1) refer patients to other programs or outpatient consultations with other members of the multidisciplinary health team and/or 2) add them to a support group (e.g., Diabetes Club, a Conversation Map^4^ group, or a self-care group). The duration of each outpatient consultation is 30 minutes.

### Multidisciplinary integrated patient care

MIDE patient care includes individualized patient assessment (outpatient consultations) with a multidisciplinary team of specialists in nursing, nutrition, social work, dental care, psychology, and physical activity. During each consultation, the specialist provides brief, personalized interventions that emphasize basic concepts of diabetes education and health promotion.

#### Nursing.

Nursing activities include educating PWD about their disease, with an emphasis on self-monitoring; teaching them how to self-administer insulin, and what to do in the case of hypoglycemia; measuring/recording vital signs and capillary HbA1c; and screening for microalbuminuria. All MIDE outpatient consultations include some type of nursing intervention.

#### Nutrition.

The nutritionist collects and records diet history and anthropometric measurements; carries out a nutrition assessment; designs a personalized food plan; educates patients (explaining how to estimate fats and carbohydrates, read labels, calculate calories, determine glycemic levels, use the equivalence system, etc.); and provides supports on nutrition-related issues in the group outpatient consultations. MIDE participants receive four nutrition outpatient consultations per year.

#### Social work.

The social work staff carry out the patient’s social assessments and are the link between the MIDE program and the patient’s family members and social network. Social workers also convene group sessions; coordinate the education/training model to promote health and patient empowerment; and keep records on program participants, issues addressed in the group sessions, and meeting outcomes. Each MIDE participant receives two individual interviews with the social worker per year.

#### Dental care.

The dental personnel perform stomatologic assessments; train patients in oral hygiene and techniques for self-assessment of dental status; conduct group sessions on oral care; and promote/suggest patient participation in support groups. Program participants receive an outpatient dental consultation every six months, unless there is a dental condition that warrants more frequent interventions, in which case they may also undergo a consult at a dental clinic.

#### Psychology.

The psychology staff assesses the mental health status of the patient; promotes the transfer of responsibility for health and medical control of diabetes to the patient; and encourages a positive attitude toward the disease, based on the underlying premise that the patient is not a “diabetic” but rather a “person with diabetes.” The psychology staff also directs patients to self-care groups, and provides support to group sessions. Program participants receive one psychology outpatient consultation each month.

#### Physical activity.

The physical activity specialist is responsible for promoting individual or group physical activity plans. This health professional conducts group exercise sessions and contributes to self-care and Conversation Map meetings. Program participants meet with the physical activity professional once per month.

### Education program

The MIDE education program—known by the acronym “AMARTE VA”^5^—provides training for both health care personnel (with a focus on vocational skills in diabetes) and patients (with a focus on empowerment and self-care).

#### Training of health care personnel.

The training curriculum for the multidisciplinary health team covers 1) orientation; 2) continuing education on program development, adherence to program operating criteria, and managerial activities (online or blended learning); and 3) vocational training. Vocational training (professionalization) covers three categories: 1) diabetes education; 2) diabetology; and 3) facilitating organization, development, and group action on type 2 diabetes. The purpose of the training is to develop health personnel’s skills in promoting patient empowerment and self-care. Therefore, the training curriculum focuses on 1) a patient-centered approach, 2) attitudinal change, and 3) collaborative learning (i.e., a horizontal relationship between the patient and health personnel). The training is designed to develop skills in boosting motivation, a sense of self-efficacy, and effective collaboration.

#### Empowerment of patients.

To initiate this process, the multidisciplinary health team launches a “diabetes literacy” campaign to promote knowledge about diabetes and help patients develop a lifestyle that enables them to achieve a level of self-care (“self-care empowerment”). This process also includes educating the patient about health care; encouraging his/her active participation and decision-making in his/her diabetes care; ensuring ISSSTE support of the patient for medical control of the disease, and to prevent complications; and consolidating and expanding social networks to support patients and their families.

To initiate the “literacy in diabetes” process, the multidisciplinary health team 1) uses “motivational interviewing”^6^ to detect patient ambivalence regarding behavior or life habits that can affect their health and 2) leads an education program designed to develop patient skills in seven self-care behaviors identified by the American Association of Diabetes Educators (AADE) (18–22). At least two sessions (workshops) of 1–2 hours each are developed to cover each of the seven behaviors. The workshops are designed to help patients acquire a new skill applicable in their daily life to promote a positive change in their health. Conversation Map is also used.

### Patient medical outcomes

The main indicator of program success is MetC (HbA1c < 7%), for all program participants. Patient HbA1c is tested four times per year and values are compared with previous values (baseline, or the last value recorded at least three months from baseline). Changes from baseline in triglycerides and total cholesterol (the second most important medical indicator) are also measured, along with fasting/postprandial and casual plasma glucose levels, and microalbuminuria, according to the criteria of the Clinical Practice Guideline for diagnosis, outpatient control goals, and timely reference of prediabetes and diabetes mellitus type 2 in adults in the first level of care (23). The percentage of reduction for each MetC indicator is calculated as the difference between the basal absolute value at the beginning of the program and the last value reported during patient follow-up, divided by the base value and multiplied by 100.^7^

Other medical outcomes measured included the number of IDs and HEs per 12-month period. Patient information for both variables was obtained through a survey. The reduction in IDs and HEs was estimated by subtracting the number reported after 12 months of program participation from the numbers recorded for the patients at baseline.

### Statistical analysis

The results of the data analysis were expressed as absolute and relative frequencies (with their corresponding 95% confidence intervals (CIs)) and compared using Yates’ chi-square test. The percentages of the reductions for the metabolic indicators were compared using one-way analysis of variance (ANOVA). Means were compared using Tamhane’s T2 post-hoc test. The probability value was adjusted using the Bonferroni correction. A probability value < 0.01 was considered statistically significant.

### Ethical considerations

This study was conducted in accordance with good clinical practices (GCPs), as defined by Mexican law, and the Helsinki Declaration for research using human subjects. The study protocol was approved by an ISSSTE research ethics review committee (*Delegación Regional Zona Norte*). To guarantee the anonymity of the patients, identifier numbers were used.

## RESULTS

**FIGURE 1.**
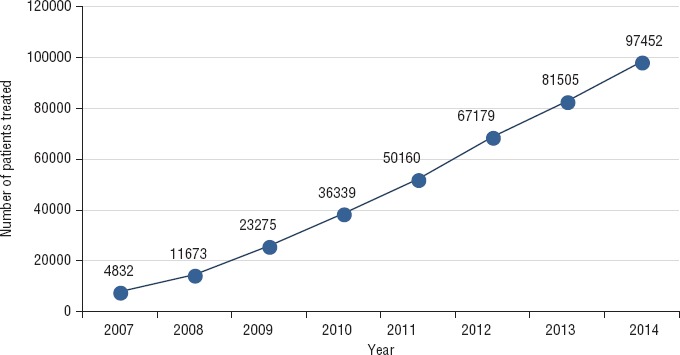
Number of patients participating in the Integrated Management of Diabetes in Stages (MIDE) program,^a^ Mexico, 2007–2014

***Source:*** Prepared by the authors using information from the MIDE national administrative database.

^a^*Manejo Integral de la Diabetes por Etapas,* a primary health care program for diabetes patients at Institute for Social Security and Services for State Workers (*Instituto de Seguridad y Servicios Sociales de los Trabajadores del Estado*, ISSSTE) hospital clinics and family medical clinics nationwide.

A record total of 97 452 PWD participated in the MIDE program between 2007 and 2014 (Figure 1). The results obtained from the program, measured with the process, structure, and impact indicators described above, were unprecedented at the ISSSTE. Table 1 defines the indicators, by type, and lists the respective study results.

### MIDE modules

**FIGURE 2.**
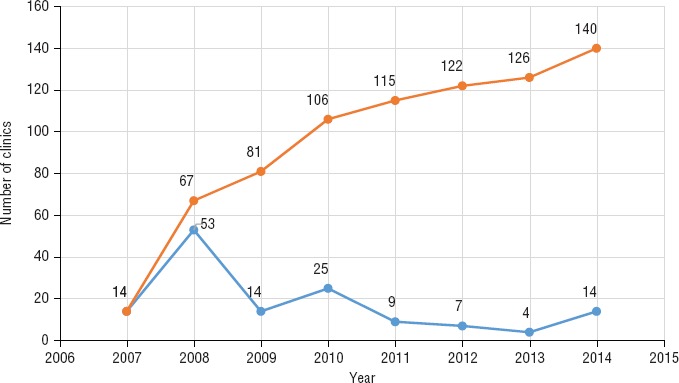
Number of clinics incorporated into the Integrated Management of Diabetes in Stages (MIDE) program (i.e., MIDE modules installed in ISSSTE primary care units) cumulatively (red) and by year (blue), Mexico, 2007–2014

***Source:*** Prepared by the authors using information from the national MIDE administrative database. *Manejo Integral de la Diabetes por Etapas,* a primary health care program for diabetes patients at Institute for Social Security and Services for State Workers (*Instituto de Seguridad y Servicios Sociales de los Trabajadores del Estado,* ISSSTE) hospital clinics and family medical clinics nationwide.

Over the seven-year study period, a total of 140 ISSSTE clinics had MIDE modules installed in their primary health care units (Figure 2). Based on the number of modules added per year (an average of 20), this study found that installation of comprehensive multidisciplinary health care services in primary health care systems is a very gradual process. The accreditation process has also been gradual, with a total of 23 or 16.4% meeting MIDE program criteria thus far.

### Multidisciplinary integrated patient care

The main result of multidisciplinary integrated patient care is education. Diabetes patients participating in the MIDE program learned to 1) assume and exert responsibility for their own health, take control of their disease management, and help prevent complications by making informed and assertive decisions; 2) evaluate the efficiency of their decisions; and 3) take advantage of the resources provided by the ISSSTE primary health care program.

### Education program

A total of 1 117 diplomas were awarded to 826 health professionals as part of the “training of health professionals” (vocational/professionalization) component. A total of 192 professionals were trained as diabetes educators, 156 physicians were trained as diabetologists, and 151 were trained as facilitators in organization, development, and group action on type 2 diabetes. The “patient empowerment” training component resulted in accreditation of 2 613 PWD as “patient experts in diabetes.”

**TABLE 1.** Definitions and results for process, structure, and impact indicators used to evaluate the Integrated Management of Diabetes in Stages (MIDE) program,^a^ Mexico, 2007–2014

Indicator	Definition	Main result
Process		
Metabolic variables		
Metabolic control (MetC)	Proportion (%) of patients with glycated hemoglobin (HbA1c) < 7.0%	62%^b^
Average HbA1c reduction per patient	% decrease over study period	25% reduction
Average triglyceride reduction per patient	% decrease over study period	31% reduction
Average total cholesterol reduction per patient	% decrease over study period	11% reduction
Structure		
Medical services		
MIDE modules	Number installed in primary health care units of ISSSTE clinics	140 modules installed
Education program		
Training of health professionals	Health professionals awarded diplomas in three areas of vocational training	1 117 diplomas awarded to 826 health professionals: 192 as diabetes educators 156 as diabetologists 151 as facilitators in organization, development, and group action on type 2 diabetes
Patient empowerment	Patients accredited as “experts in diabetes”	2 613 patients accredited
Impact		
Medical outcomes		
Average number of illness days per patient	% decrease over study period	38% reduction
Average number of hospitalization episodes per patient	% decrease over study period	41% reduction

a *Manejo Integral de la Diabetes por Etapas*, a primary health care program for diabetes patients at Institute for Social Security and Services for State Workers (*Instituto de Seguridad y Servicios Sociales de los Trabajadores del Estado*, ISSSTE) hospital clinics and family medical clinics nationwide.

b For the year 2013—the highest level of MetC recorded over the study period (MetC for 2014 was 60%).

***Source:*** Prepared by the authors based on the study results.

### Patient medical outcomes

**FIGURE 3.**
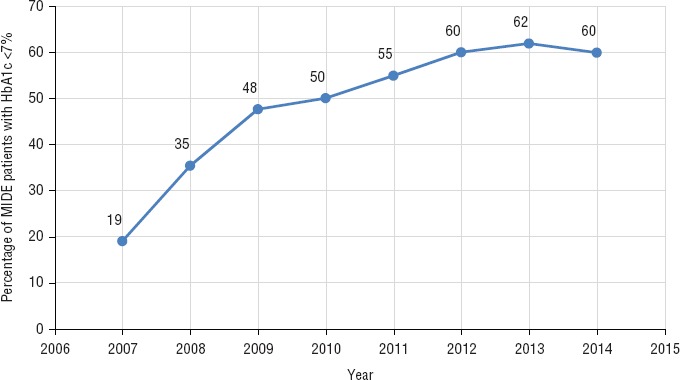
Percentage of diabetes patients with metabolic control (MetC)^a^ after 3 or more months of participation in the Integrated Management of Diabetes in Stages (MIDE) program,^b^ Mexico, 2007–2014

***Source:*** Prepared by the authors using information from the national MIDE administrative database.

^a^ Glycated hemoglobin (HbA1c) < 7%). ^b^*Manejo Integral de la Diabetes por Etapas*, a primary health care program for diabetes patients at Institute for Social Security and Services for State Workers (*Instituto de Seguridad y Servicios Sociales de los Trabajadores del Estado*, ISSSTE) hospital clinics and family medical clinics nationwide.

As shown in Table 2 and Figure 3, the proportion of MIDE program participants with controlled diabetes (HbA1c < 7.0%) increased significantly over the seven-year study period, except between 2012 and 2014. The most significant reduction (2%) occurred between 2013 and 2014 (Table 2). In addition, average levels for the three metabolic indicators (HbA1c, triglycerides, and total cholesterol) per PWD dropped by 25% (CI: 24.6–25.6); 31% (CI: 30.1–31.9); and 11% (CI: 10.2–11.5) respectively compared to baseline levels (Table 1).

For the two impact indicators (number of IDs and number of HEs per 12-month period), which were used to evaluate a very important aspect of integrated treatment of diabetes patients—quality of life—there was a reduction of 38% (CI: 37.2–38.9) and 41% (CI: 40.3–41.5) respectively (Table 1). This drop represented a significant decline in spending for the ISSSTE and improved quality of life for the patients.

**TABLE 2.** Proportion (%) of diabetes patients with metabolic control (MetC)^a^ after 3 or more months of participation in the Integrated Management of Diabetes in Stages (MIDE) program,^b^ by year, and relative change (±%), Mexico, 2007–2014

Year	2007	2008	2009	2010	2011	2012	2013	2014
% MetC (95% CI^d^)	19.0% (19.2–19.4)	35.4% (35.1–35.7)	47.7% (47.2–47.9)	50.1% (49.5–50.5)	55% (54.6–55.5)	60.1% (59.3–60.9)	62% (61.1–62.5)	60% (59.8–60.8)
Relative change (±%)								
2007 19.0% (19.2–19.4)	0	-16.4 (-16.3 to -16.5)	-28.7 (-28.6– -28.8)	-31.1 (-30.9– -31.2)	-36.0 (-35.9– -36.2)	-41.1 (-40.9– -41.2)	-43.0 (-42.9– -43.2)	-41.0 (-40.8– -41.3)
2008 35.4% (35.1–35.7)	+16.4 (16.3–16.5)	0	-12.3 (-11.8– -12.8)	-14.7 (-14.2– -15.2)	-19.6 (-19.2– -20.0)	-24.7 (-24.3– -25.1)	-26.6 (-26.2– -27.0)	-24.6 (-24.3– -24.9)
2009 47.7% (47.2–47.9)	+28.7 (28.6–28.8)	+12.3 (11.8–12.8)	0	-2.4 (-2.2– -2.6)	-7.3 (-7.0– -7.6)	-12.4 (-12.1– -12.7)	-14.3 (-14.0– -14.6)	-12.3 (-12.1– -12.5)
2010 50.1% (49.5–50.5)	+31.1 (30.9–31.2)	+14.7 (14.2–15.2)	+2.4 (2.2–2.6)	0	-4.9 (-4.7– -5.1)	-10 (-9.8 to -10.2)	-11.9 (-11.7– -12.1)	-9.9 (-9.7– -10.1)
2011 55% (54.6–55.5)	+36.0 (35.9–36.2)	+19.6 (19.2–20.0)	+7.3 (7.0–7.6)	+4.9 (4.7–5.1)	0	-5.1 (-4.9– -5.3)	-7.0 (-6.8– -7.2)	-5.0 (-4.9 to -5.1)
2012 60.1% (59.3–60.9)	+41.1 (40.9–41.2)	+24.7 (24.3–25.1)	+12.4 (12.1–12.7)	+10 (9.8–10.2)	+5.1 (4.9–5.3)	0	-1.9 (-1.8– -2.0)	+0.1 (0.010–0.11)^e^
2013 62% (61.1–62.5)	+43.0 (42.9–43.2)	+26.6 (26.2–27.0)	+14.3 (14.0–14.6)	+11.9 (11.7–12.1)	+7.0 (6.8–7.2)	+1.9 (1.8–2.0)	0	+2.0 (1.9–2.1)
2014 60% (59.8–60.8)	+41.0 (40.8–41.3)	+24.6 (24.3–24.9)	+12.3 (12.1–12.5	+9.9 (9.7–10.1)	+5.0 (4.9–5.1)	-0.1 (-0.010– -0.11)^e^	-2.0 (-1.9– -2.1)	0

***Source:*** Prepared by the authors using information from the national MIDE administrative database.

a Glycated hemoglobin (Hb1Ac) < 7%).

b *Manejo Integral de la Diabetes por Etapas*, a primary health care program for diabetes patients at Institute for Social Security and Services for State Workers (*Instituto de Seguridad y Servicios Sociales de los Trabajadores del Estado*, ISSSTE) hospital clinics and family medical clinics nationwide.

c All *P* values calculated using one-way analysis of variance (ANOVA). Means compared using the Tamhane T2 post-hoc test. *P* values < 0.001 except for the comparison between the years 2012 and 2014.

d Confidence interval.

e *P* value > 0.001.

## DISCUSSION

This study showed that after participation in the MIDE program, a large proportion of PWD in Mexico improved their status with respect to glucose, HbA1c, total cholesterol, and triglycerides, and decreased their IDs and HEs, suggesting an overall improvement in the quality of primary care provided by the ISSSTE clinics. These results are similar to results for another PEP reported by Wong et al. in Hong Kong (7) but differ from PEP results from 2015 reported by Khan et al. (24). Several studies have indicated that a PEP is associated with improved HbA1c levels compared with the results observed in patients who 1) did not participate in a PEP, 2) were treated with diet, and/or 3) were treated under the traditional health care paradigm of medicalized care (7, 25–27). The pilot study of the DESMOND program^8^ found that the reduction in program participants’ HbA1c was significant at three-month follow-up but was not significant at one- and three-year follow-up (28–30). Pillay et al. concluded that the self-care PEP evaluated in their study, which offered ≤ 10 hours of contact with a health team, provided little benefit except among persons with suboptimal or poor glycemic control, who benefited more than those with good control (31).

### Patient empowerment

Compared with the PEP studied by Wong et al., and the X-PERT (32) and DESMOND programs, Mexico’s MIDE program incorporates more elements focused on patient empowerment, such as motivational interviewing, the promotion of self-care and motivation, and the establishment of goals in coordination with the multidisciplinary health team (7, 28–30). Patient empowerment also seems to facilitate an environment conducive to attitudinal change and the incorporation of new lifestyle behaviors.

The patient empowerment focus also allows for increased investment in health through public primary health services because it includes personalized, educational communication to help ensure the proper use of resources, restore health, limit damage, and prevent disability.

MIDE program strategies empower patients by promoting 1) self-efficacy and self-confidence with respect to health; 2) horizontal (patient–physician) collaboration, which is fundamental to maintaining newly learned behaviors (11); 3) treatment adherence; and 4) a more assertive style of patient–physician communication, which allows for joint decision-making. Effective patient–physician collaboration is very important because it helps change the mindset/behavior of health professionals from medication prescribers to facilitators and agents of change. This type of collaboration is taught in the professionalization (vocational training) of the multidisciplinary health care team and is an important part of the MIDE education program.

### Self-care and MetC

The collaborative, community-based, constructivist nature of the MIDE education programs, in which patients and health personnel exchange information (knowledge, and experiences), creates a bidirectional communication process conducive to learning, and thus also helps increase patient self-care skills and “ownership” of (responsibility for) their disease management, and synergy among members of the group, who feel connected and supported. With better disease management skills, and professional support, patients are more apt to achieve MetC.

The multidisciplinary health care team promotes self-care, emphasizes the development of abilities and skills, encourages social participation and proactive decision-making, expands social networks, and is sensitized in the effective promotion of health and empowerment. Thus, the team is also a facilitator of growth of the self-care groups. This process is a perfect complement for the patient empowerment process.

### Public health model

By combining these two effective processes, MIDE is an effective public health model for addressing NCDs, and a new paradigm that can be applied and adapted in other settings. More important, it is an appropriate model for generating the required structural changes in public health institutions, and can be used in care for other NCDs, so would most likely result in an increase in patient coverage. Obtaining optimal results would require standardizing the model’s managerial and clinical processes to benefit the entire population covered by the ISSSTE.

### Limitations and strengths

Study limitations include the lack of analysis of 1) the influence of factors associated with access to social security, given that the entire study sample was covered by the ISSSTE, and 2) social and cultural mechanisms that can affect the medical care process (e.g., different housing conditions and economic levels). Strengths of the MIDE program include 1) the involvement of many different actors and tools to support the education programs for both patients and health care personnel, and 2) the patient-centered approach to the treatment of diabetes, which includes tools for motivating changes in behavior in patients and involving their family members and social networks.

### Recommendations

Future research should address the following questions: 1) “What are the factors or determinants of an effective education process?” and 2) “What characteristics inherent to the teaching-learning process are linked to the patients, the health personnel, and the health care system?” Additional studies could also evaluate the benefit of integrated medical services in terms of reducing diabetes complications (7, 33). The authors also recommend 1) empowerment training for patients, and training for multidisciplinary health care personnel on integrated treatment, and 2) the development, organization, and management of groups designed to promote self-care and medical adherence, incorporated in general public health policy. Finally, patient empowerment could be studied further to identify the factors that influence the development of health care professionals ad hoc to the epidemiologic panorama of each region.

## CONCLUSIONS

The results of this study show that installing integrated, specially designed medical services in primary health care systems; providing educational activities for health care personnel, and an empowerment program for patients and their families; and encouraging the full participation of multidisciplinary health care teams improves MetC indicators and reduces illness and hospital stays in people with diabetes.

## Acknowledgments

The authors would like to thank Dr. Joel Rodríguez-Saldaña for the initial implementation of the MIDE program (Mexico City).

## Funding.

Funding was provided by Mexico’s Instituto de Seguridad y Servicios Sociales de los Trabajadores del Estado (ISSSTE) through the Secretaria de Hacienda y Crédito Público (SHCP).

## Disclaimer.

Authors hold sole responsibility for the views expressed in the manuscript, which may not necessarily reflect the opinion or policy of the RPSP/PAJPH or the Pan American Health Organization (PAHO).

## Footnotes

3 Treating or defining human conditions or problems as medical conditions.

4 Interactive patient-centered tool for diabetes education, designed for use with small groups (6–10 persons) (13–17).

5 “A” for healthy eating (*alimentarme saludablemente*); “MA” for being active (*manteniéndome active*); “R” for reducing risk (*reduciendo riesgos*); “T” for taking medication (*tomando mis medicamentos*); “E” for problem-solving (*encontrando soluciones*); “V” for Monitoring (*vigilando mis valores*); and “A” for healthy coping (*adaptándome saludablemente*).

6 A technique that facilitates recognition of risk behaviors and allows the multidisciplinary health team to identify potential areas for increasing patient motivation in self-care.

7 For example, if HbA1c = 7.7 at the beginning of treatment, and the last HbA1c during follow-up = 5.0, the difference in HbA1c = 2.7 (7.7 − 5.0), and the percentage of reduction in HbA1c = 35.1% (2.7/7.7 * 100).

8 Diabetes education and self-management for ongoing and newly diagnosed PWD.
